# Development of the First Tractable Genetic System for Parvimonas micra, a Ubiquitous Pathobiont in Human Dysbiotic Disease

**DOI:** 10.1128/spectrum.00465-22

**Published:** 2022-04-13

**Authors:** Dustin L. Higashi, Sean McGuire, Yasser M. Abdelrahman, Zhengzhong Zou, Hua Qin, David Anderson, Elizabeth A. Palmer, Jens Kreth, Justin Merritt

**Affiliations:** a Department of Restorative Dentistry, Oregon Health and Science University, Portland, Oregon, USA; b Department of Pediatric Dentistry, Oregon Health and Science University, Portland, Oregon, USA; c Department of Microbiology and Immunology, Faculty of Pharmacy, Cairo University, Giza, Egypt; d Department of Molecular Microbiology and Immunology, Oregon Health and Science University, Portland, Oregon, USA; McGill University

**Keywords:** Gram-positive bacteria, *Parvimonas micra*, genetics, natural competence

## Abstract

Parvimonas micra is a Gram-positive obligate anaerobe and a typical member of the human microbiome. P. micra is among the most highly enriched species at numerous sites of mucosal dysbiotic disease and is closely associated with the development of multiple types of malignant tumors. Despite its strong association with disease, surprisingly little is known about P. micra pathobiology, which is directly attributable to its longstanding genetic intractability. To address this problem, we directly isolated a collection of P. micra strains from odontogenic abscess clinical specimens and then screened these isolates for natural competence. Amazingly, all of the P. micra clinical isolates exhibited various levels of natural competence, including the reference strain ATCC 33270. By exploiting this ability, we were able to employ cloning-independent methodologies to engineer and complement a variety of targeted chromosomal genetic mutations directly within low-passage-number clinical isolates. To develop a tractable genetic system for P. micra, we first adapted renilla-based bioluminescence for highly sensitive reporter studies. This reporter system was then applied for the development of the novel Theo+ theophylline-inducible riboswitch for tunable gene expression studies over a broad dynamic range. Finally, we demonstrate the feasibility of generating *mariner*-based transposon sequencing (Tn-seq) libraries for forward genetic screening in P. micra. With the availability of a highly efficient transformation protocol and the current suite of genetic tools, P. micra should now be considered a fully genetically tractable organism suitable for molecular genetic research. The methods presented here provide a clear path to investigate the understudied role of P. micra in polymicrobial infections and tumorigenesis.

**IMPORTANCE** Parvimonas micra is among the most highly enriched species at numerous sites of mucosal dysbiotic disease and is closely associated with numerous cancers. Despite this, little is known about P. micra pathobiology, which is directly attributable to its longstanding genetic intractability. In this study, we provide the first report of P. micra natural competence and describe the only tractable genetic system for this species. The methods presented here will allow for the detailed study of P. micra and its role in infection and tumorigenesis.

## INTRODUCTION

Parvimonas micra is a Gram-positive obligate anaerobe from the largely uncharacterized *Tissierellia* class of the *Firmicutes* phylum. Initially grouped within the *Peptostreptococcus* genus, a taxonomic reclassification revealed its distinction within the *Firmicutes* (1, 2). P. micra is a common constituent of the microbiomes from multiple mucosal sites in the body, including the oral cavity, gastrointestinal tract, respiratory system, and female urogenital tract. Epidemiological studies implicate P. micra as a major human pathobiont. For example, it is both an exceptionally common and highly enriched species in numerous studies of different mucosal inflammatory diseases (3). In addition, P. micra is among the most common sources of Gram-positive anaerobic cocci (GPAC) sepsis. It is also one of the most abundant species in numerous types of systemic abscesses, and it exhibits a surprisingly strong association with a variety of malignant tumors (4–7). In fact, P. micra has even been proposed as a discriminating biomarker for colorectal cancer, gastric cancer, and oral cancer (4, 8, 9). In the oral cavity, P. micra exists as a low-abundance species during oral health but is highly enriched in periodontitis lesions and infected root canals and is especially prevalent in polymicrobial odontogenic abscesses (10–15).

The mechanisms by which P. micra contributes to human health and disease remain largely enigmatic, as there is a severe paucity of literature describing its molecular genetics (7). Furthermore, this organism has been historically challenging to identify in clinical microbiology laboratories, largely due to its fastidious nature and slow growth rate (3). Reports suggest that P. micra frequently exhibits pathogenic synergism with other pathobiont members of the microbiome. A recent *in vitro* study illustrated how P. micra can augment the growth of Porphyromonas gingivalis and enhance its production of secreted proteolytic gingipains (16). *In vivo* studies demonstrated an enhanced transmissibility of pus generated from P. micra/*Prevotella* coinfections compared to that of their respective mono-infections (17). P. micra also coaggregates with the well-characterized oral pathobionts Treponema denticola and Fusobacterium nucleatum (18, 19).

Despite its strong association with a broad diversity of mucosal inflammatory diseases, P. micra remains vastly understudied, largely due to its genetic intractability. Only a single report of targeted mutagenesis has ever been described for this organism, and this was accomplished via electroporation of a suicide vector (20). The lack of follow-up genetic studies underscores the significant challenges associated with genetically manipulating P. micra. Such problems have severely restricted the options for detailed mechanistic studies of P. micra pathobiology. To address this problem, we present the first efficacious P. micra genetic system, which is founded upon a newly discovered natural competence ability in this organism. By exploiting this ability, we were able to apply cloning-independent methodologies to engineer a variety of targeted chromosomal genetic modifications directly within low-passage-number clinical isolates. The efficacy of this P. micra transformation protocol further supported the development of the first tractable genetic system for this species. With the genetic toolbox presented here, a complete molecular genetic system is in place to reliably interrogate P. micra pathobiology. These strategies applied here may also have potential utility for the study of other genetically intractable members of the microbiome.

## RESULTS

### Discovery of natural competence among P. micra clinical isolates.

**FIG 1:**
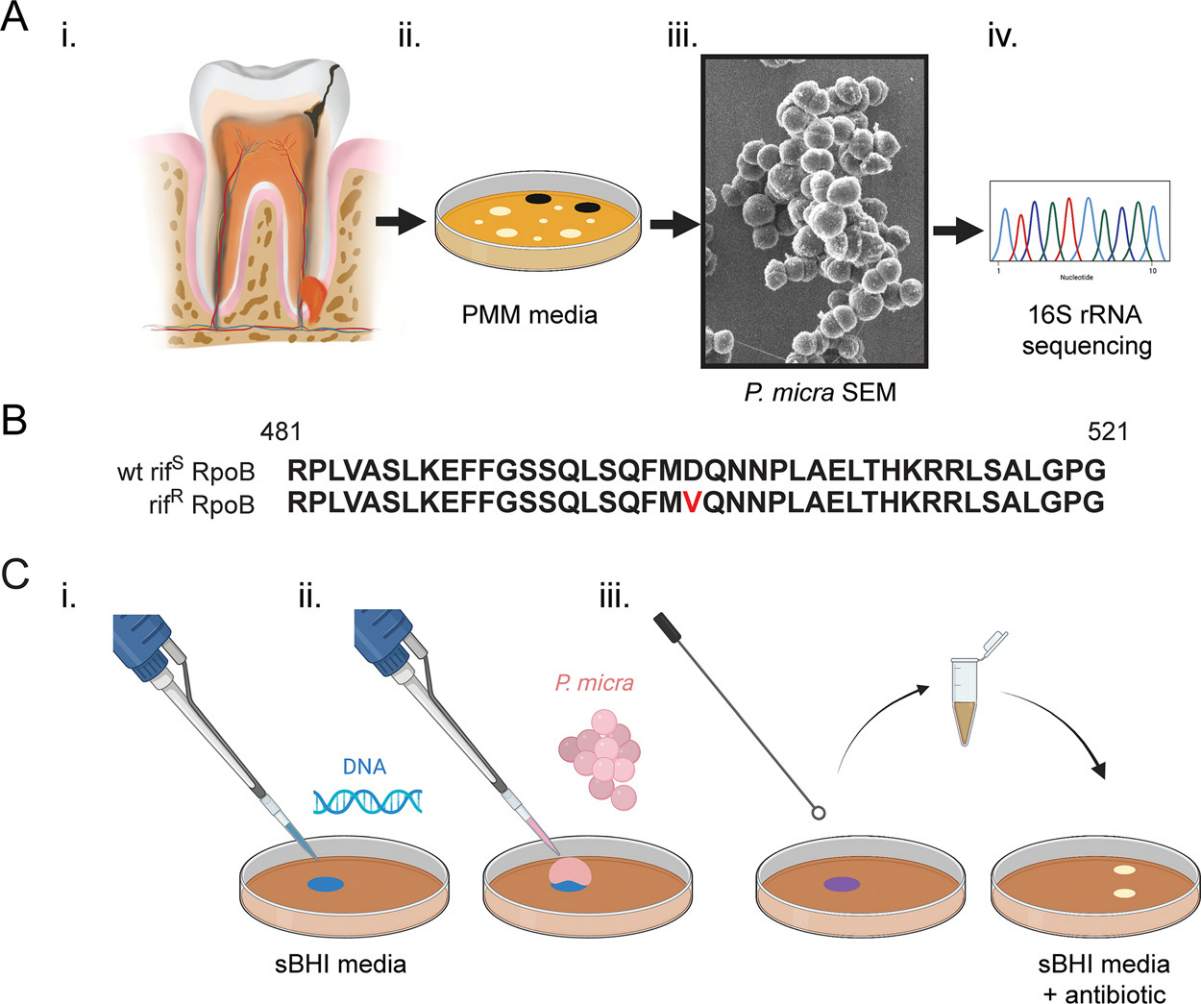
Parvimonas micra clinical isolation scheme and transformation assay. (A) (i) Odontogenic abscess samples were collected into anaerobic transport medium and (ii) grown on selective/differential agar PMM. Putative colonies were (iii) confirmed morphologically and (iv) submitted for 16S rRNA sequencing to verify species identity. (B) Wild-type strain ATCC 33270 (wt rif^S^) was grown on rifampin containing sBHI medium to isolate a spontaneous rifampin resistance strain (rif^R^). The deduced amino acid sequences of the wild-type rif^S^ and rif^R^ RpoB proteins are shown. The rif^R^ mutation is shown in red font. (C) Plate transformation assays were performed by the addition of (i) DNA and (ii) P. micra to sBHI agar. (iii) After 24 h of growth, bacteria were collected into sBHI medium and plated on sBHI medium supplemented with the appropriate antibiotic.

Recently, Liu and Hou described the only known report of targeted mutagenesis in P. micra (20). This study employed a cloning-based methodology to create a mutagenesis construct introduced via electrotransformation of a suicide plasmid. Due to the limited effectiveness of P. micra electroporation, we were interested to determine whether a more reliable and efficacious transformation approach could be developed using natural competence. To test this, we first isolated a collection of low-passage-number clinical strains directly from odontogenic abscess specimens (Fig. 1A). Following tooth extraction, abscess pus samples were collected into prereduced transport medium (21) and then spread onto selective/differential agar Peptostreptococcus micros medium (PMM) (12). Candidate P. micra colonies were selected based upon the accumulation of black precipitate, then subsequently examined morphologically by scanning electron microscopy, and finally confirmed with 16S rRNA sequencing (Fig. 1A).

We next generated a selectable marker for use in DNA transformation assays by isolating a spontaneous rifampin-resistant mutant of the wild-type reference strain ATCC 33270. A resulting rifampin-resistant (rif^R^) colony of ATCC 33270 was verified by sequence analysis of the *rpoB* gene encoding the beta subunit of RNA polymerase. A point mutation in this gene confirmed a D501V substitution in RpoB (Fig. 1B), which coincides with rifampin resistance mutations detected in other bacterial species (22, 23). Next, genomic DNA (gDNA) was extracted from both the wild-type strain ATCC 33270 (rif^S^) and the rifampin-resistant mutant of ATCC 33270 (rif^R^) and then transformed into our collection of wild-type P. micra clinical isolates using a transformation protocol (Fig. 1C) similar to that previously employed for Veillonella parvula (24). With this approach, bacteria are exposed to transforming DNA through multiple growth stages and a wide range of cell densities, and the reaction occurs in an environment analogous to that of a biofilm. Surprisingly, we observed natural competence from the majority of strains tested, with particularly high transformation efficiencies in a number of isolates (Table 1). These results confirmed that P. micra is capable of developing natural competence.

**TABLE 1:** Transformation frequency and fold increase of Parvimonas micra isolates^*a*^

Parvimonas micra isolate	ATCC 33270 gDNA (rif^R^)	Transformation frequency (rif^R^/total CFU) (×10^−4^)^*b*^	Fold increase (over background)
A28	+	1.73 ± 0.34*	752
	−	0.0023 ± 0.0005	
A42	+	0.33 ± 0.12*	236
	−	0.0014 ± 0.0002	
A11	+	0.26 ± 0.06*	263
	−	0.00099 ± 0.00014	
ATCC 33270	+	0.075 ± 0.0029*	36
	−	0.0021 ± 0.0022	
A3	+	0.013 ± 0.00013*	8
	−	0.0016 ± 0.00043	
A1	+	0.0040 ± 0.0012^n.s.^	1
	−	0.0035 ± 0.0014	

a Transformation frequency is expressed as the number of rifampin-resistant bacteria/total CFU. +, indicates rif^R^ and −, indicates WT gDNA. Values are averaged from triplicate determinations ± SD. Statistical analysis was performed using a two-tailed Student’s *t* test.

b “*” indicates significance (*P* < 0.05) relative to WT gDNA control. “n.s.” indicates no significance (*P* > 0.60).

### Cloning-independent targeted mutagenesis.

**FIG 2:**
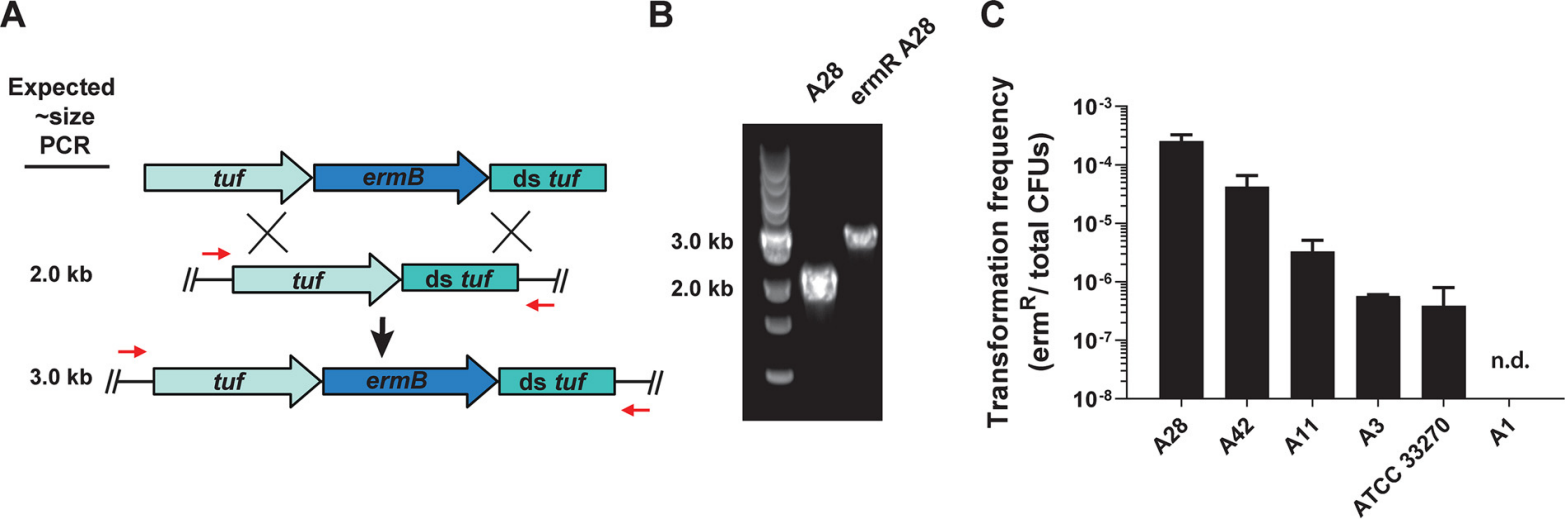
Insertion of resistance cassette into P. micra clinical isolates. (A) Mutagenesis construct (top) was inserted into the P. micra chromosome through homologous recombination (middle) yielding erythromycin-resistant transformants (bottom). The primers used for PCR verification of genotypes are illustrated by red arrows. (B) Insertion of the erythromycin resistance cassette (∼1 kb) was confirmed by PCR for a selected transformant (ermR A28). (C) Transformation frequencies of P. micra wild-type strains. Transformation frequencies are expressed as the ratio of erythromycin-resistant CFU to total CFU. Values represent the averages from triplicate independent determinations ± standard deviation (SD). “n.d.” indicates a transformation efficiency below the detection limit of the assay.

Following our discovery of P. micra natural competence, it was of interest to determine whether this ability could be exploited for engineering targeted mutations in P. micra. For this, we chose the *ermB* erythromycin resistance cassette as a selectable marker (25). An allelic insertion mutagenesis construct was created by Gibson assembly of PCR amplicons containing *ermB* flanked by 1 kb homologous fragments of the EF-Tu-encoding gene *tuf* and its downstream locus (Fig. 2A). The insertion of the *ermB* cassette immediately upstream of the native *tuf* terminator ensured its robust expression. Transformation of this construct into the clinical strain A28 resulted in the insertion of *ermB* immediately downstream of the *tuf* open reading frame (ORF) due to a double-crossover allelic exchange event (Fig. 2A and B). Next, replicate constructs were generated for each isolate and then transformed into their respective strains, including the reference strain ATCC 33270. In each of these reactions, background erythromycin resistance was undetectable, whereas 4 out of 5 isolates, including ATCC 33270, exhibited detectable levels of natural competence (Fig. 2C).

### Comparison of construct homology versus transformation efficiency.

**FIG 3:**
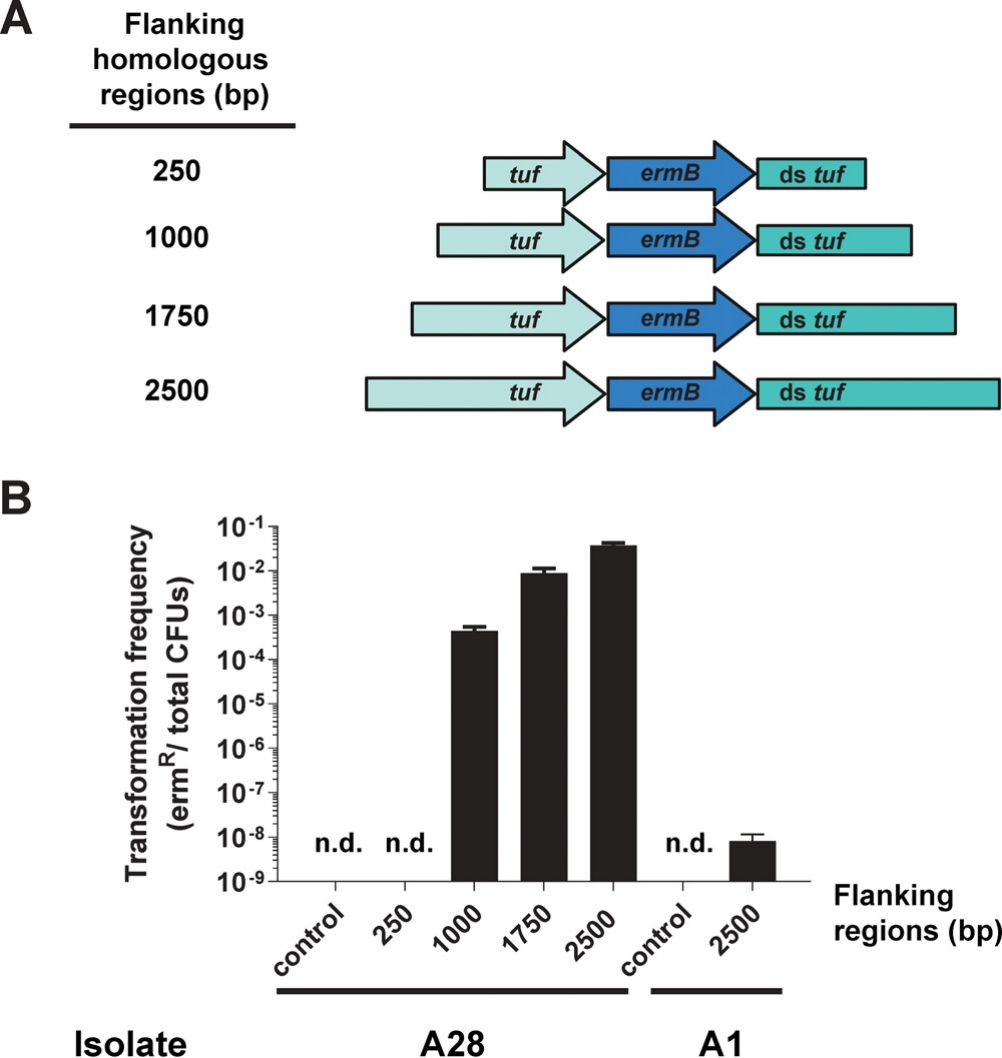
Comparison of homology versus transformation efficiency in P. micra. (A) Mutagenesis constructs were generated with flanking regions of various sizes. (B) Wild-type clinical isolates A28 and A1 were transformed with the mutagenesis constructs illustrated in panel A. Values are averaged from triplicate independent determinations ± SD. “n.d.” indicates a transformation efficiency below the detection limit of the assay.

In an attempt to further bolster the transformation efficiencies of our isolates, we next tested a variety of mutagenesis constructs (Fig. 3A) to examine the correlation between the sizes of the homologous DNA fragments and the resulting transformation efficiencies (26, 27). For this, we focused on isolate A28, which displayed the highest level of natural competence using 1-kb homologous fragments (Fig. 2C). No transformants were detected in the negative-control samples or when using mutagenesis constructs containing homologous flanking regions of 250 bp (Fig. 3B). An increase in the size of the homologous fragments from 1,000 bp to 1,750 bp resulted in a >100-fold increase in transformation efficiency. A further increase in homologous fragment length from 1,750 bp to 2,500 bp resulted in only an additional ∼15% increase in transformation efficiency, which suggested that transformation rates were approaching their maximum. Given the significant impact of larger homologous flanking regions upon the transformation rates of strain A28, we subsequently reexamined the transformation-negative strain A1 (Fig. 2C) using 2.5-kb homologous flanking arms. With this larger construct, it was then possible to detect reliable, albeit low, levels of transformation in this strain (Fig. 3B). These results demonstrate that the size of construct homology plays a significant role for natural transformation efficiency in P. micra, with homologous arms of ≥1,750 bp being optimal for maximal rates of transformation.

### Targeted mutations of *recA* in P. micra.

**FIG 4:**
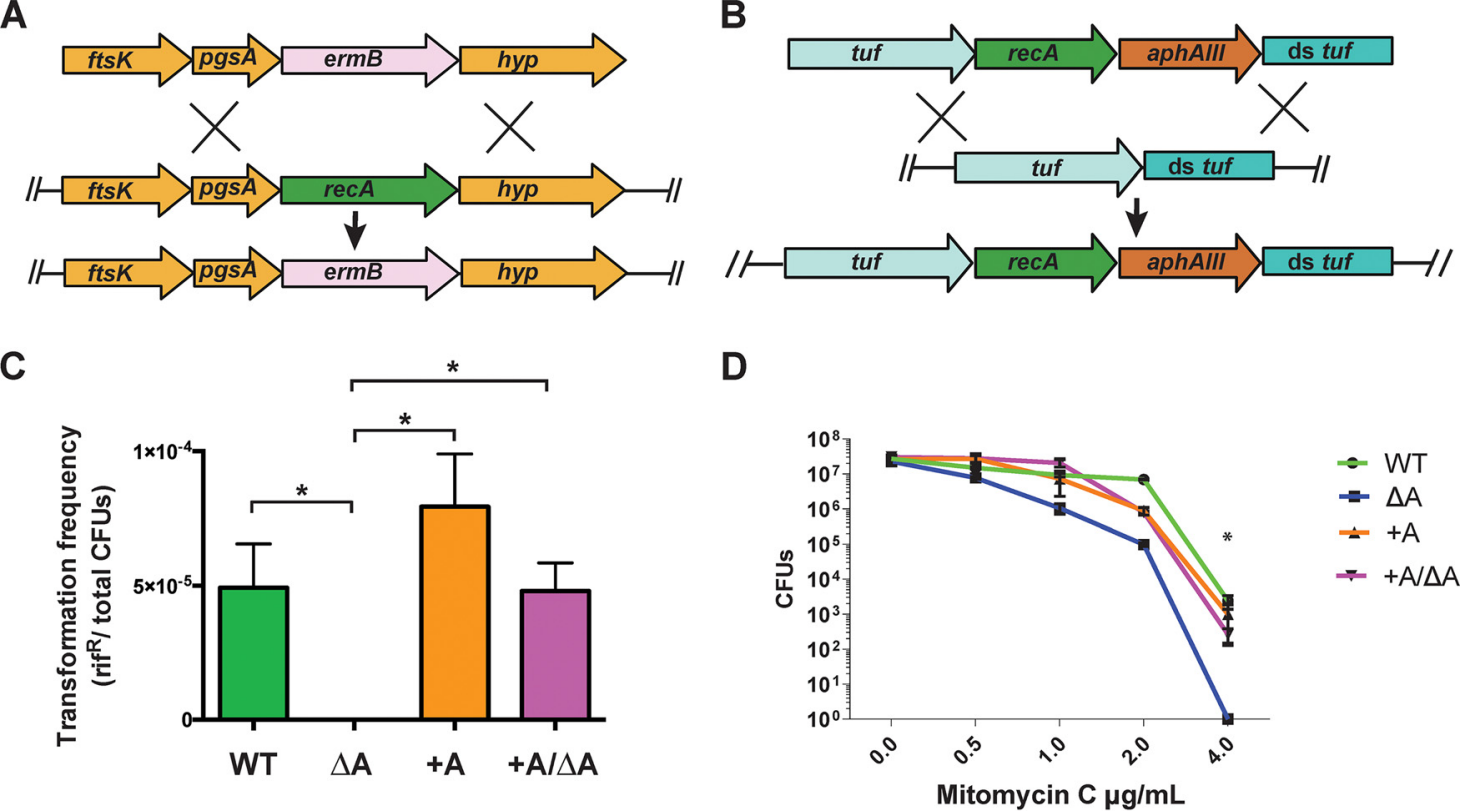
Targeted mutations of *recA* in P. micra. (A) The *recA* deletion construct (top) was inserted into the P. micra A28 chromosome through homologous recombination (middle), resulting in allelic replacement of the *recA* ORF with an *ermB* cassette (bottom). (B) A *recA* knock-in mutagenesis construct (top) was inserted into the P. micra chromosome through homologous recombination (middle), resulting in the insertion of the *recA* ORF and *aphAIII* cassette downstream of EF-Tu (bottom). The Δ*recA* mutation was complemented by moving this mutation into the *recA* knock-in background. (C) Transformation frequency of wild-type A28 (WT), A28 Δ*recA* mutant (ΔA), double *recA-*expressing knock-in mutant (+A), and complemented Δ*recA* mutant (+A/ΔA). Transformation frequency is expressed as the ratio of rifampin-resistant bacteria to total CFU. (D) Mitomycin C survival curves. All values are averaged from triplicate independent determinations ± SD. For transformation assays, comparisons are indicated by brackets. For MMC experiments, comparisons are presented relative to ΔA. Statistical analysis was performed using a two-tailed Student’s *t* test. *, *P* < 0.05.

We were next interested to determine whether our P. micra transformation protocol could be used for targeted allelic replacement mutagenesis of *recA*. RecA is a nonessential DNA-dependent ATPase that plays a central role in homologous DNA recombination and repair (28, 29). Given the requirement for homologous recombination during allelic replacement mutagenesis, it was anticipated that a *recA* mutant would exhibit a natural transformation defective phenotype, preventing subsequent mutagenesis using a marked linear DNA construct. Similar to other bacteria, we also anticipated that a *recA* mutant would exhibit a highly increased sensitivity to DNA damage. We tested this in strain A28 by first transforming a *recA* deletion construct comprised of an erythromycin resistance cassette (*ermB*) flanked by 1 kb DNA fragments homologous to the upstream and downstream regions surrounding *recA* (Fig. 4A). This resulted in a double-crossover allelic exchange deletion mutant in which *recA* was replaced with *ermB* (Fig. 4A). Since the transformation defects resulting from a *recA* mutation would likely preclude subsequent genetic complementation, it was necessary to first create an ectopically expressed *recA* knock-in mutant to serve as a recipient strain for a deletion of the native *recA* gene. A mutagenesis construct comprised of the *recA* open reading frame (ORF) followed by a kanamycin resistance cassette (*aphAIII*) (25) was ligated to 2.5 kb segments of the *tuf* gene and its downstream sequence (Fig. 4B). After transforming this construct into strain A28, a double-crossover event resulted in the insertion of an ectopic copy of the *recA* ORF and *aphAIII* directly downstream of *tuf* (Fig. 4B). In this double *recA*-expressing background, we subsequently deleted the native *recA* gene using the same *recA* deletion construct as that described above (Fig. 4A). Consequently, the resulting erythromycin/kanamycin-resistant transformant harbored a deletion of the endogenous *recA* while also expressing an ectopic copy of *recA* together with the *tuf* gene as part of an artificial operon (Fig. 4B). We subsequently compared the transformation efficiencies of these *recA* mutant strains using a PCR-generated amplicon of the rif^R^ *rpoB* gene previously generated in the strain ATCC 33270 (Fig. 1B). The double *recA-*expressing strain (+A) and the complemented *recA* deletion strain (+A/ΔA) both exhibited transformation efficiencies similar to that of the wild type (WT), whereas the Δ*recA* deletion strain (ΔA) yielded no detectable levels of natural competence (Fig. 4C). As an independent verification of the *recA* deletion phenotype, we further tested these strains for sensitivity to the genotoxic agent mitomycin C (MMC). As expected, the Δ*recA* strain (ΔA) displayed a greater sensitivity to MMC at all of the tested dosages (0.5 to 4 μg mL^−1^), with a >3-log reduced survival rate observed at the highest MMC dosage (Fig. 4D). In agreement with the transformation results, both the single and double *recA*-expressing strains behaved similarly to the wild type. Taken together, these results demonstrate the successful implementation of a highly efficient P. micra mutagenesis protocol that can be employed for both targeted gene deletion and genetic complementation using naturally transformed PCR products.

### Creation of a renilla luciferase reporter.

**FIG 5:**
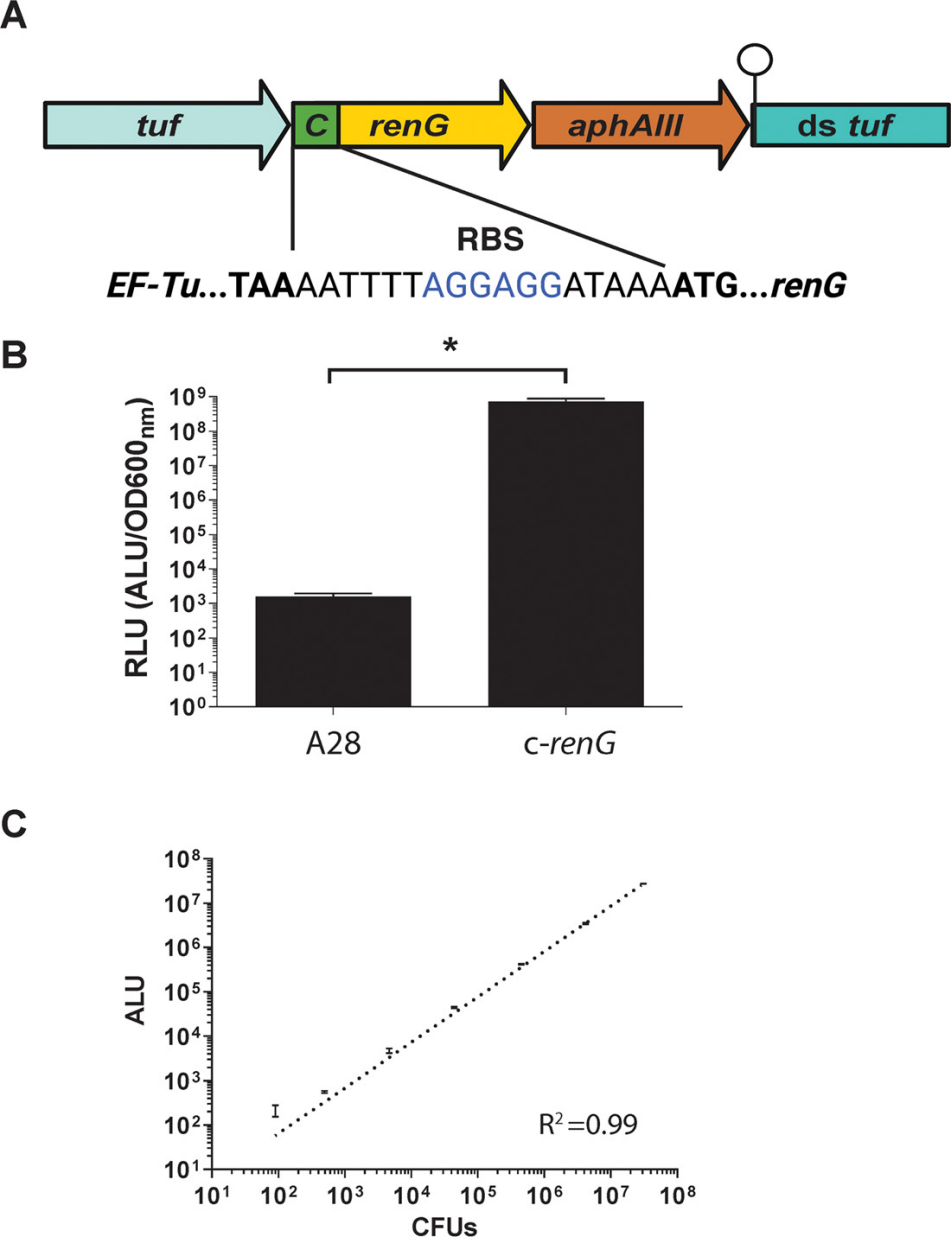
Green renilla luciferase (RenG) expression in P. micra. (A) Genomic context and sequence of a constitutively expressed *renG* transcription fusion to the *tuf* gene. The *tuf* gene Rho-independent terminator is indicated by a ball and stick icon. (B) Luciferase activity was measured in wild-type strain A28 and the luciferase reporter strain (c-*renG*). Normalized luciferase activity (RLU) was calculated by dividing raw luciferase values by the culture OD_600_ values. (C) Correlation between viable cell counts (CFU) and luciferase reporter values in the constitutive luciferase reporter strain c-*renG* (R^2^ = 0.99). All data points represent the average values ± SD from triplicate independent determinations. Statistical analysis was performed using a two-tailed Student’s *t* test. *, *P* < 0.05.

The high quantum yield and low background of luciferase-based reporter assays can provide a rapid and precise measure of gene expression over an exceptionally wide dynamic range. The superior signal-to-noise ratio of bioluminescent reporters has also supported noninvasive biophotonic imaging studies of mice orally infected with human oral streptococci (30). To determine whether similar luciferase reporters would also function in P. micra, we developed a constitutive green renilla luciferase expression strain by creating a transcriptional fusion of the *tuf* gene, green renilla (*renG*) luciferase, and a kanamycin resistance cassette (*aphAIII*). This construct was inserted immediately downstream of the *tuf* ORF but upstream of the endogenous *tuf* terminator (Fig. 5A). Green renilla luciferase (RenG) has been successfully employed in several species of oral Streptococcus and displays a signal intensity much higher than that of firefly luciferase (30). Luciferase assays of the P. micra reporter strain demonstrated extremely high levels of bioluminescence that were >5 logs above the background readings of the unmodified parent strain A28 (Fig. 5B). To gauge the detection limits and precision of the luciferase reporter readings in this strain, we next examined the correlation between population size and luciferase bioluminescence values. A dilution series of the reporter exhibited an extremely strong linear correlation to bioluminescence readings, with precise reporter values detectable in as few as 10^2^ to 10^3^ total CFU (Fig. 5C). This suggests that luciferase reporter assays can be accurately performed within an exceptionally wide range of P. micra cell densities.

### Tunable gene expression system in P. micra.

**FIG 6:**
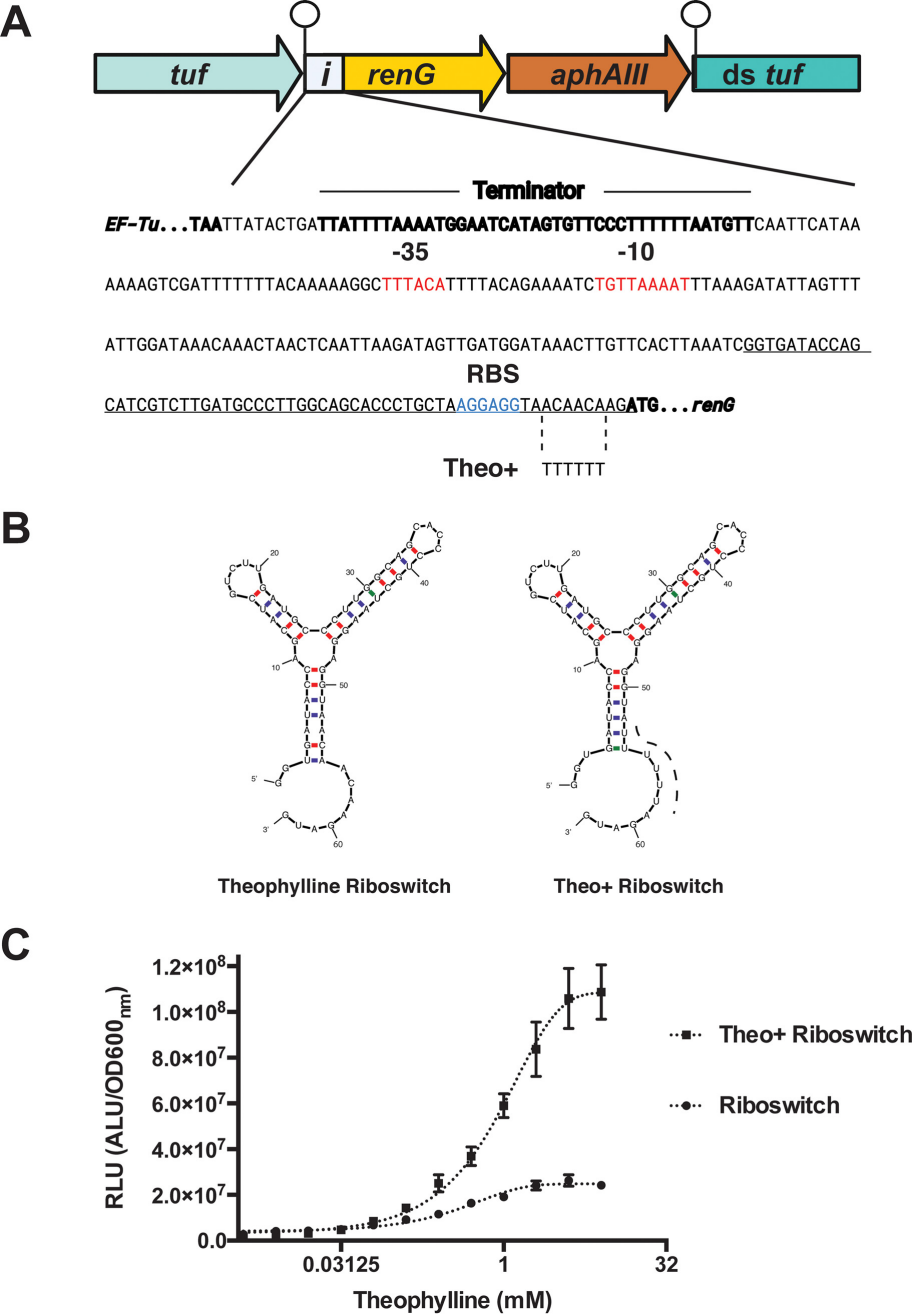
Regulated gene expression system in P. micra. (A) Genomic context (top) and corresponding sequence (bottom) for an inducible luciferase reporter strain (i-*renG*) in P. micra. The *tuf* gene Rho-independent terminator is indicated by a ball and stick icon. The sequences are shown for the promoter region of the luciferase reporter, with the original theophylline riboswitch (underlined) and Theo+ riboswitch (dashed lines, bottom) indicated. Promoter elements are shown in red font, while the ribosomal binding site is in blue font. (B) The mFold webserver (http://www.unafold.org/mfold/applications/rna-folding-form.php) was used to predict the secondary structures of the original (left image) and Theo+ (right image) theophylline riboswitches. (C) The original and Theo+ riboswitches were compared for their abilities to regulate luciferase reporter activity over a range of theophylline concentrations. Normalized luciferase activity (RLU) was calculated by dividing raw luciferase values by the respective OD_600_ values. Data points represent the average values ± SD from triplicate independent determinations.

The efficacy of the green renilla luciferase reporter system subsequently provided an avenue to further develop a regulated gene expression system in P. micra. Ideally, such systems would display a low basal expression along with strong and tunable levels of induction. We first tested the xylose-inducible expression cassette Xyl-S1 developed for oral *Streptococcus* species (31). However, this yielded only nominal xylose-dependent regulation in P. micra. Therefore, we were curious whether a posttranscriptional regulatory system, such as those of ligand-inducible riboswitches (32), might perform better. To test this, we inserted a theophylline riboswitch (32) between the *renG* ORF and an ectopic copy of the constitutively expressed *rpoB* promoter. Like the previous *renG* reporter construct (Fig. 5A), we similarly inserted the riboswitch-controlled reporter construct immediately after the *tuf* ORF (Fig. 6A). However, for this construct, we also included a second ectopic copy of the endogenous *tuf* transcription terminator upstream of the construct to minimize transcriptional read-through from the *tuf* gene (Fig. 6A). The secondary structure of the theophylline riboswitch is shown in Fig. 6B. This strain was assayed for luciferase reporter activity over a range of theophylline concentrations, and we observed a titratable dose response curve with ∼9-fold dynamic range (Fig. 6C). This result is highly consistent with previous studies employing this riboswitch in other species (32). Encouraged by the inducibility of this riboswitch in P. micra, we sought to improve upon its dynamic range by introducing a series of point mutations into the riboswitch. Since the theophylline riboswitch is predicted to fold into a series of stable stem-loop structures (Fig. 6B), we were curious whether the inclusion of a poly-uracil tract immediately after the terminal stem-loop would create a Rho-independent terminator-like element to synergize with the ribosome binding site sequestration already present within the riboswitch, further increasing the magnitude of riboswitch regulation. A similar dual regulatory mechanism has been reported for the flavin mononucleotide riboswitch found on the *ribB* gene of Escherichia coli (33). Furthermore, mFOLD predictions suggested that these mutations could be introduced without triggering obvious changes to the overall riboswitch secondary structure (Fig. 6B). After examining the dose response curve of the modified riboswitch reporter strain, we indeed observed a greatly enhanced response to theophylline, resulting in a >70-fold dynamic range (Fig. 6C). Together, these results demonstrate the utility of *renG-*encoded luciferase as a sensitive reporter protein in P. micra as well as a riboswitch-based tunable gene expression system that exhibits low basal expression and robust inducibility.

### Development of *in vitro* transposon mutagenesis for Tn-seq studies.

**FIG 7:**
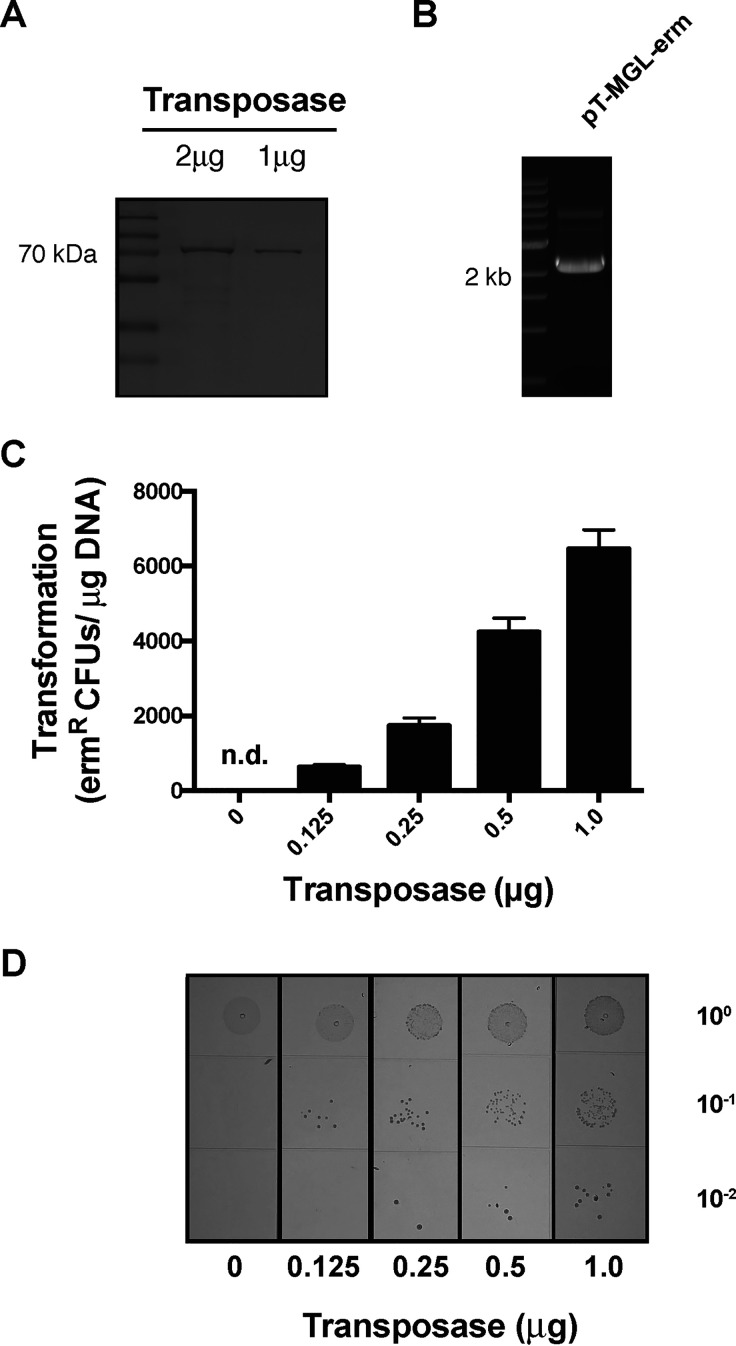
*In vitro mariner* transposon mutagenesis in P. micra. (A) Purification of recombinant MarC9 transposase and (B) the mini-*mariner* transposon plasmid pT-MGL-erm. (C) One microgram of P. micra gDNA was mutagenized using 0.125, 0.25, 0.5, and 1.0 μg of recombinant MarC9 transposase with a fixed quantity of the mini-*mariner* transposon. These reactions were subsequently transformed into wild-type P. micra strain A28. The transformation results are shown on the graph and by (D) growth on erythromycin supplemented media. Data are expressed as the average number of transposon mutants/μg of gDNA. Values are averaged from triplicate independent determinations ± SD. “n.d.” indicates a transformation efficiency below the detection limit of the assay.

Transposon sequencing (Tn-seq) is a powerful forward genetic screening tool that has been successfully employed for genetic fitness studies of a number of pathogens (34) and pathobiont microbiome species (25, 35). Given the high level of natural competence observed in P. micra, we were curious to determine the feasibility of implementing *in vitro* transposon mutagenesis using recombinant MarC9 transposase and the mini-*mariner* transposon commonly used in Tn-seq studies (Fig. 7A and B) (25, 34, 36). Following *in vitro* transposon mutagenesis of P. micra gDNA, the reactions were transformed directly into the wild-type strain A28. With this approach, we readily obtained >6,000 transposon mutants per microgram gDNA (Fig. 7C and D). Considering that the P. micra genome is <2 Mb, a single *in vitro* mutagenesis reaction with several micrograms of P. micra gDNA would be sufficient to create densely saturated libraries of transposon insertions that are directly compatible with Tn-seq protocols. Overall, P. micra should be highly amenable to Tn-seq studies of its pathobiology.

## DISCUSSION

P. micra is a notable pathobiont in the oral cavity due to its strong association with a variety of inflammatory oral diseases. P. micra is also commonly identified as a major constituent of acute and chronic infections at numerous other sites in the human body, including the abdomen, chest, spine, brain, blood, skin, and urogenital tract (37, 38). Many P. micra infections have been described as polymicrobial, though a number of reports have implicated it as the sole source of infection as well (39, 40). Interestingly, the presence of P. micra has also been strongly associated with multiple types of cancer. Numerous reports have identified a major enrichment of P. micra in colorectal tumors (5, 41–43), while others have reported an association of P. micra with oral and gastric cancers (4, 43, 44). Given the substantial body of evidence for its role in human disease, the development of new genetic tools for P. micra is an essential step to reveal its unknown pathogenic mechanisms.

The P. micra genome contains a number of genes with putative functions essential for natural competence in organisms such as Haemophilus influenzae and Bacillus subtilis, including predicted traffic NTPases (PilB, ComGA) and pilin/pseudopilins (PilA, ComGC). This led us to consider the possibility that P. micra may also be naturally transformable. To the best of our knowledge, our demonstration of P. micra natural competence is not only a first for the species but is also likely the first report of natural competence within the entire *Tissierellia* class, which is a large subgroup in the *Firmicutes* phylum composed largely of microbial dark matter. The use of natural competence for DNA transformation has numerous advantages over artificial methods, such as chemical treatments (e.g., CaCl_2_) and electroporation (45). These approaches are also difficult to optimize for many fastidious anaerobes like P. micra, due to their sensitivity to excessive handling. Natural transformation can also be more efficient than artificial methods, as naturally transformed DNA enters the bacterial cytosol single-stranded (46). Unlike duplex DNA introduced by artificial means, single-stranded DNA (ssDNA) is largely resistant to degradation by most bacterial restriction enzymes (47, 48). Furthermore, ssDNA can serve as a more efficient substrate for homologous recombination than duplex DNA. The recombinase RecA is specifically activated by the presence of ssDNA to form presynaptic filaments, which in turn promote RecA to scan for homologous sequences, induce synapse formation, and ultimately mediate recombination (49). In our study, all of the P. micra isolates tested displayed some level of natural competence (Fig. 2C and Fig. 3B), though it is currently unclear why some strains exhibited higher transformation rates than others. A number of factors could contribute to these disparities, such as strain-specific differences in the expression of competence related genes, variable responses to competence-specific environmental cues, and/or an altered expression of transformation-limiting bacterial factors (e.g., nucleases) (50). Our results demonstrate that some of these limiting factors can be mitigated by increasing the lengths of the homologous fragments contained on the mutagenesis constructs (Fig. 3B) (51). Based on this, we speculate that endogenous exonuclease activity is likely to be a major determinant of the transformation efficiency observed with naturally transformed linear DNA constructs. However, it is also possible that longer segments of homology simply lead to higher rates of recombination.

After observing robust natural competence in P. micra, we examined the feasibility of creating *recA* deletion mutants. We chose *recA* as a test case due to its anticipated and easily measured phenotypes. As expected, the *recA* mutant exhibited a total loss of detectable natural transformability as well as an MMC-sensitive phenotype (Fig. 4C and D). We were also able to complement these phenotypes by employing allelic exchange to insert the *recA* ORF immediately downstream of the constitutively expressed *tuf* gene, creating an artificial *tuf-recA* operon (Fig. 4B). This further illustrates a straightforward approach for genetic complementation without requiring a shuttle vector.

The subsequent expression of luciferase in P. micra led us to consider its use as a reporter for the development of a regulated gene expression system. Previously, we developed a highly efficient xylose induction system (XylR/O) that functioned in several oral *Streptococcus* species (31). Repressor/operator-based systems such as XylR/O (xylose) and LacR/O (IPTG) are contingent upon the uptake of inducer molecules as well as the heterologous expression of the corresponding repressor proteins. These can be of limited utility for bacterial species in which repressor protein expression or inducer permeability may be limiting factors. Indeed, our attempts to develop a XylR/O system in P. micra were largely unsuccessful. This led us to consider an alternative approach using a riboswitch-based regulatory system for posttranscriptional control of gene expression. Using a synthetic theophylline riboswitch (32), we demonstrated the potential utility of theophylline induction in P. micra, achieving up to an ∼9-fold maximum induction of luciferase activity (Fig. 6C). In an attempt to further improve upon its performance, we introduced a series of riboswitch point mutations that were predicted to create a Rho-independent terminator-like structure in the absence of theophylline (Fig. 6A and B). Further studies would be required to confirm whether the mutant riboswitch truly functions in transcription termination. Regardless, these mutations were able to further increase the dynamic range of the theophylline riboswitch by nearly an order of magnitude (Fig. 6C). This new version that we now refer to as the Theo+ riboswitch yielded low basal expression while providing robust and tunable inducibility. In addition to genetic complementation, this induction system is likely applicable for studies of both essential and toxic genes. It may also be useful for controlling target transcript levels and the timing of gene expression during infection studies.

Tn-seq is a powerful forward genetic screening tool used for genome-level assessments of genetic fitness. Based upon the efficacy of P. micra natural transformation, we predicted that it would be well suited for *in vitro mariner*-based transposon mutagenesis. Considering that the Himar transposase specifically targets TA dinucleotides (36), the high A+T content of the P. micra genome (>70% A+T) makes it especially well suited for Tn-seq analysis with Himar/*mariner*. The availability of a high-efficiency natural competence protocol also circumvents the requirement for complex *in vivo* transposition methods employing conjugation, temperature-sensitive plasmids, and ectopically expressed transposases. Based upon the results shown in Fig. 7C and D, the current protocol should easily achieve the mutagenesis thresholds required for high-density Tn-seq analysis. Thus, when combined with the other genetic tools described in this study, one can now reliably perform nearly all of the molecular genetic approaches required to characterize P. micra pathobiology. As such, P. micra should also be added to the list of genetically tractable oral microbes.

## MATERIALS AND METHODS

### Isolation of *Parvimonas* from clinical odontogenic abscess samples.

Clinical sample collection and strain isolation protocols were reviewed by the Oregon Health and Science University Institutional Review Board (IRB) prior to the initiation of the study and deemed to be not human subjects research. All clinical specimens were collected by clinicians in the OHSU Pediatric Dental clinic using materials generated during routine treatment procedures. Specimens were deidentified of all protected health information (PHI) as part of the collection protocol. Clinical specimens were derived from a pediatric cohort undergoing tooth extraction due to odontogenic abscesses.

Abscess samples were collected using Dacron swabs and immediately placed in prereduced transport medium (21). Abscess samples were vortexed and plated onto agar PMM (12). Plates were incubated in an anaerobic chamber (85% N_2_, 10% CO_2_, 5% H_2_) at 37°C for 4 to 7 days. Bacterial colonies producing a black precipitate were passaged on fresh PMM plates. Candidate isolates were imaged by microscopy for the expected cellular morphologies and then screened by PCR using the primers 16SFOR/REV. Sequence identification of 16S rRNA was confirmed using the expanded Human Oral Microbiome Database (www.homd.org) (52).

### Bacterial strains and growth conditions.

P. micra cultures were maintained in supplemented brain heart infusion medium (sBHI), which consisted of base brain heart infusion medium (BHI; Gibco, Gaithersburg, MD) containing 0.5% wt/vol yeast extract (Fisher, Fair Lawn, NJ), 0.005% wt/vol hemin (Sigma, St. Louis, MO), 0.001% wt/vol menadione (Sigma, St. Louis, MO), and 0.05% wt/vol cysteine (Sigma, St. Louis, MO). sBHI medium and cultures were maintained in anaerobic conditions (85% N_2_, 10% CO_2_, 5% H_2_) at 37°C. For antibiotic selection, sBHI agar plates were supplemented with the following: rifampin (5 mg L^−1^), kanamycin (300 mg L^−1^), and erythromycin (15 mg L^−1^).

To isolate spontaneous rifampin-resistant mutants, the P. micra reference strain ATCC 33270 was plated on sBHI agar containing rifampin (5 mg L^−1^). A rifampin-resistant mutant (rif^R^) was screened by PCR using the primers rpoB-1240FOR and rpoB-2095REV. Sequence analysis of this conserved region of *rpoB* confirmed an A-to-T transversion resulting in an aspartic acid-to-valine substitution at amino acid 501 of RpoB.

### Electron microscopy.

Bacteria were grown overnight in sBHI on indium-tin-oxide (ITO) coverslips and fixed in 2.5% glutaraldehyde plus 2.5% formaldehyde in sodium cacodylate buffer (pH 7.4). Samples were rinsed in buffer, postfixed in 2% osmium tetroxide for 30 min, rinsed in water, dehydrated in a graded ethanol series, and critical point dried in a Leica CPD300. Samples were mounted on aluminum stubs with a carbon tab and coated with 8 nm carbon using a Leica ACE600 coater. Imaging was performed on a ThermoFisher Scientific Helios NanoLabG3 DualBeam scanning electron microscope.

### Extraction of genomic DNA from P. micra.

Genomic DNA (gDNA) was purified using a phenol-chloroform extraction procedure (53). Bacteria were harvested from sBHI agar plates, suspended in STES buffer (0.5 M NaCl, 0.02 M EDTA, 0.2 M Tris-HCl [pH 8], 20 mg mL^−1^ lysozyme), and incubated at 55°C for 2 h. SDS was added to a final concentration of 1% followed by the addition of proteinase K (0.1 mg mL^−1^) and incubation at 55°C for 1 h. One volume of phenol/chloroform/isoamyl alcohol (25:24:1; Fisher, Fair Lawn, NJ) was added to the suspension and mixed gently. After centrifugation at 16,000 × *g*, the aqueous layer was collected and treated with RNase (50 μg mL^−1^) at 37°C for 2 h followed by a second extraction with phenol/chloroform/isoamyl alcohol. A 1:10 volume of 3 M sodium acetate (pH 5.5) and 3 volumes of ice-cold ethanol were added and incubated at −20°C for 24 h. DNA was pelleted by centrifugation at 16,000 × *g* at 4°C for 30 min and subsequently washed with ice-cold ethanol (70%). The DNA pellet was dried in a laminar flow hood and resuspended in double-distilled water (ddH_2_0).

### DNA transformation experiments.

Transformation assays were performed similarly as described previously (24). Briefly, P. micra was grown on sBHI agar for 72 h. Cells were harvested from plates, suspended into liquid sBHI, and adjusted to an optical density at 600 nm (OD_600_) of 0.4. Unless otherwise indicated, a total of 1 μg of gDNA (dissolved in 20 μL of ddH_2_0) was spotted onto sBHI agar plates. After complete absorption of the gDNA into the agar, 20 μL of each bacteria suspension was pipetted over the DNA spots and absorbed. Plates were incubated in anaerobic conditions at 37°C for 24 h. Following the incubation period, cells were then harvested into 50 μL of sBHI and vortexed. Serial dilutions were spread onto sBHI agar plates containing the appropriate antibiotic as well as on nonselective sBHI plates to determine total CFU counts. Transformation efficiency was calculated as the ratio of antibiotic-resistant transformants to total CFU. For allelic replacement mutagenesis and genetic complementation, transformations were performed similarly as described above.

### P. micra *ermB* insertion mutagenesis constructs.

Primers and mutagenesis constructs were designed using Serial Cloner software (https://serial-cloner.en.softonic.com). All primers used in this study are listed in Supplemental File 1. All PCR amplifications were performed using Phusion high-fidelity polymerase (Fisher, Fair Lawn, NJ). For insertion mutagenesis constructs, an erythromycin resistance cassette (*ermB*) was inserted directly downstream of the *tuf* gene to ensure robust expression. The *ermB* cassette, originally isolated from Staphylococcus aureus, has shown diverse utility in both Gram-positive and Gram-negative bacteria (54). This cassette naturally lacks a transcription terminator. Therefore, the *ermB* cassette containing its endogenous promoter and ORF was amplified using primers ermR-FOR/REV and the plasmid pJY4164 as a PCR template (25). Upstream homologous regions were generated using the primer pair tuf-FOR/REV for all isolates. For downstream homologous regions, the primer pair down-tuf-FOR/REV was used with isolates A1, A3, and A28, while down-tufFORa/tuf-REV was used for strains A11 and ATCC 33270. gDNA from each respective P. micra strain was used as the PCR template. All amplicons were screened for size, column purified (Qiagen, Germantown, MD), and assembled using Gibson assembly master mix (NEB, Ipswich, MA) per the manufacturer’s instructions. The assembled construct was amplified by PCR using the primer pair tuf-FOR/down-tuf-REV, screened for size, and column purified. Transformation assays using these constructs were performed as described above. The primer pair tuf-FOR*/down-tuf-REV* was used to screen transformants for *ermB* insertion.

To generate *ermB* insertion constructs with flanking regions of various sizes, gDNA from an *ermB* insertion mutant (ermR A28) was used as the template. The primer pairs tuf-250-FOR/REV, tuf-FOR/down-tuf-REV, tuf-1750-FOR/REV, and tuf-2500-FOR/REV were used to generate homologous fragments of 250 bp, 1.0 kb, 1.75 kb, and 2.5 kb, respectively. For transformation assays, a molar equivalent to 1 μg of the 1.0 kb homologous flank construct was used for all constructs. Transformation assays with these constructs were performed as described above.

### P. micra *recA* deletion and genetic complementation.

For construction of the *recA* deletion mutant, a mutagenesis construct consisting of an *ermB* cassette flanked by homologous upstream and downstream regions to *recA* was assembled. The *ermB* cassette was amplified using the primer pair ermR-FOR/REV and plasmid pJY4164 as the PCR template. Upstream and downstream homologous regions to *recA* were generated using the primer pairs up-recA-FOR/REV and down-recA-FOR/REV, respectively, with strain A28 gDNA serving as the PCR template. All amplicons were screened for size, column purified, and assembled using Gibson assembly master mix. The assembled construct was amplified by PCR using the primer pair up-recA-FOR/down-recA-REV, screened for size, and column purified. Transformants were selected on agar plates supplemented with erythromycin.

To generate the *recA* knock-in strain, a construct was made containing the *recA* ORF along with a kanamycin resistance cassette *aphAIII* inserted immediately downstream of the *tuf* gene. The *aphAIII* gene was originally isolated from Enterococcus faecalis and has exhibited broad utility in numerous bacterial genetic systems (55). To accomplish this, the PCR fragments homologous to *tuf* and its downstream sequence were generated using the primer pairs tuf-FOR/tuf-REVa and down-tuf-FORb/tuf-2500-REV, respectively, with A28 gDNA serving as the PCR template. The *recA* gene was amplified using the primer pair recA-FOR/REV and A28 gDNA as the template. The kanamycin resistance cassette *aphAIII* was amplified with its endogenous transcription terminator using the primer pair kan-FOR/REV with plasmid pWVTK as the template (25). All amplicons were screened for size, column purified, and assembled using Gibson assembly master mix. The assembled construct was PCR amplified using the primer pair tuf-FOR/tuf-2500-REV and then column purified. Transformants were selected on agar plates supplemented with kanamycin. A complemented Δ*recA* mutant strain was generated by transforming the *recA* knock-in strain with the *recA* deletion construct described above. Transformants were selected on agar plates supplemented with kanamycin and erythromycin.

To compare transformation efficiencies of the various *recA* mutants, PCR amplicons were generated with the primer pair rpoB-1240FOR/2095REV using gDNA from the spontaneous P. micra rifampin-resistant strain described above. PCR products were screened for size and column purified. Transformation assays were performed as described above using sBHI ± rifampin. For mitomycin C (Sigma, St. Louis, MO) sensitivity experiments, each strain was suspended in sBHI at an OD_600_ of 1.0 and then serial dilutions were plated on sBHI agar plates containing various dosages of MMC (0 μg mL^−1^, 0.5 μg mL^−1^, 1 μg mL^−1^, 2 μg mL^−1^, and 4 μg mL^−1^). Plates were incubated in an anaerobic chamber at 37°C for 72 h.

### Green renilla luciferase expression in P. micra.

To express green renilla luciferase in P. micra, a construct was assembled containing the *renG* ORF along with a kanamycin resistance cassette *aphAIII* inserted immediately downstream of the *tuf* gene. PCR fragments homologous to *tuf* and its downstream sequence were generated using the primer pairs tuf-2500-FOR/tuf-REVa and down-tuf-FORb/tuf-2500-REV, respectively, with A28 gDNA as the template. A PCR fragment of *renG* was generated using the primer pair renG-RBS-FOR/renG-REV with gDNA from strain brsRM-renG as the template (25). The kanamycin resistance cassette *aphAIII* was amplified using the primer pair kan-FOR/REV and plasmid pWVTK as the template. All amplicons were screened for size, column purified, and assembled using Gibson assembly master mix. The assembled construct was amplified by PCR using the primer pair tuf-2500-FOR/REV, screened for size, and column purified. The amplicon was transformed into wild-type P. micra strain A28 and the transformants were selected on agar plates supplemented with kanamycin.

### Construction of P. micra-inducible luciferase strain.

For inducible expression of renilla luciferase in P. micra, a construct containing the *tuf* gene rho-independent terminator followed by a *rpoB* promoter and riboswitch preceding *renG* followed by a kanamycin cassette was inserted directly downstream of the *tuf* gene. PCR fragments homologous to *tuf*, including its terminator and its downstream sequence, were generated using the primer pairs tuf-2500-FOR/tuf-53-REV and down-tuf-FORb/tuf-2500-REV, respectively, using strain A28 gDNA as the template. The riboswitch regulatory region was PCR amplified with the primer pair ribo-FOR/REV using a synthetic riboswitch DNA fragment (IDT, Coralville, IA) as the template (see Supplemental File 1). The *renG* gene was amplified with the primer pair renG-FOR/REV using gDNA from strain brsRM-renG. The kanamycin resistance cassette was amplified using the primer pair kan-FOR/REV and plasmid pWVTK as the template. All amplicons were screened for size, column purified, and assembled using Gibson assembly master mix. The resulting construct was PCR amplified using the primer pair tuf-2500-FOR/REV, screened for size, and column purified. This product was transformed into wild-type P. micra isolate A28, and transformants were selected on agar plates supplemented with kanamycin.

For the Theo+ riboswitch variant, a construct was generated using the primer pairs tuf-2500-FOR/theo-plus-ribo-REV and theo-plus-renG-FOR/tuf-2500REV using gDNA from the inducible mutant described above. PCR products were assembled using Gibson assembly master mix and amplified using the primer pair tuf-2500-FOR/REV. Transformants were selected on sBHI agar plates containing kanamycin.

### Luciferase assays.

RenG expression strains were grown for 72 h on sBHI agar plates supplemented with kanamycin. Bacteria were suspended in liquid sBHI medium with a range of theophylline concentrations (0 mM, 0.0078 mM, 0.0156 mM, 0.03125 mM, 0.0625 mM, 0.125 mM, 0.25 mM, 0.5 mM, 1 mM, 2 mM, 4 mM, and 8 mM) at an OD_600_ of 0.1 and then incubated in an anaerobic chamber at 37°C for 20 h. Coelenterazine-h solution (Prolume, Pinetop, AZ) was added to each sample (3.4 μg mL^−1^), and luciferase activity was measured on a Promega Glomax Discover luminometer. Optical densities were measured immediately after measuring luciferase activity for normalization. Normalized activity was expressed as relative light units (RLU), which is the luminescence value/OD_600_.

### Generation of a transposon insertion library in P. micra.

A transposon library was generated in P. micra strain A28 using a previously described protocol (34). Briefly, *in vitro* transposon mutagenesis was performed by combining 1 μg of gDNA from isolate A28, 1 μg of the *mariner* transposon-containing vector pT-MGL-erm (25), and various amounts of the MarC9 transposase (0 μg, 0.125 μg, 0.25 μg, 0.5 μg, and 1 μg). The mixture was incubated for 30°C for 1 h followed by a 75°C incubation for 10 min and then incubation on ice. The DNA was precipitated with ethanol and transposon junctions were subsequently repaired. The resulting transposon reactions were transformed directly into P. micra isolate A28. Serial dilutions were plated on nonselective sBHI agar plates for total counts, while transposon mutants were selected on sBHI agar plates supplemented with erythromycin.

### Statistical analysis.

All statistical analyses were performed using GraphPad Prism software to calculate significance via two-tailed Student's *t* test. Statistical significance was assessed using a cutoff value of *P* < 0.05.

## References

[1] Tindall BJ, Euzeby JP. 2006. Proposal of *Parvimonas* gen. nov. and *Quatrionicoccus* gen. nov. as replacements for the illegitimate, prokaryotic, generic names *Micromonas* Murdoch and Shah 2000 and *Quadricoccus* Maszenan et al. 2002, respectively. Int J Syst Evol Microbiol 56:2711–2713. doi:10.1099/ijs.0.64338-0. 17082417

[2] Murdoch DA, Shah HN. 1999. Reclassification of *Peptostreptococcus magnus* (Prevot 1933) Holdeman and Moore 1972 as *Finegoldia magna* comb. nov. and *Peptostreptococcus micros* (Prevot 1933) Smith 1957 as *Micromonas micros* comb. nov. Anaerobe 5:555–559. doi:10.1006/anae.1999.0197.

[3] Murphy EC, Frick IM. 2013. Gram-positive anaerobic cocci–commensals and opportunistic pathogens. FEMS Microbiol Rev 37:520–553. doi:10.1111/1574-6976.12005. 23030831

[4] Coker OO, Dai Z, Nie Y, Zhao G, Cao L, Nakatsu G, Wu WK, Wong SH, Chen Z, Sung JJY, Yu J. 2018. Mucosal microbiome dysbiosis in gastric carcinogenesis. Gut 67:1024–1032. doi:10.1136/gutjnl-2017-314281. 28765474 PMC5969346

[5] Flemer B, Warren RD, Barrett MP, Cisek K, Das A, Jeffery IB, Hurley E, O'Riordain M, Shanahan F, O'Toole PW. 2018. The oral microbiota in colorectal cancer is distinctive and predictive. Gut 67:1454–1463. doi:10.1136/gutjnl-2017-314814. 28988196 PMC6204958

[6] Saffarian A, Mulet C, Regnault B, Amiot A, Tran-Van-Nhieu J, Ravel J, Sobhani I, Sansonetti PJ, Pedron T. 2019. Crypt- and mucosa-associated core microbiotas in humans and their alteration in colon cancer patients. mBio 10. doi:10.1128/mBio.01315-19. PMC6635529 31311881

[7] Watanabe T, Hara Y, Yoshimi Y, Fujita Y, Yokoe M, Noguchi Y. 2020. Clinical characteristics of bloodstream infection by *Parvimonas micra*: retrospective case series and literature review. BMC Infect Dis 20:578. doi:10.1186/s12879-020-05305-y. 32758181 PMC7405351

[8] Yang CY, Yeh YM, Yu HY, Chin CY, Hsu CW, Liu H, Huang PJ, Hu SN, Liao CT, Chang KP, Chang YL. 2018. Oral microbiota community dynamics associated with oral squamous cell carcinoma staging. Front Microbiol 9:862. doi:10.3389/fmicb.2018.00862. 29774014 PMC5943489

[9] Yao Y, Ni H, Wang X, Xu Q, Zhang J, Jiang L, Wang B, Song S, Zhu X. 2021. A new biomarker of fecal bacteria for non-invasive diagnosis of colorectal cancer. Front Cell Infect Microbiol 11:744049. doi:10.3389/fcimb.2021.744049. 34976850 PMC8719628

[10] Colombo AP, Boches SK, Cotton SL, Goodson JM, Kent R, Haffajee AD, Socransky SS, Hasturk H, Van Dyke TE, Dewhirst F, Paster BJ. 2009. Comparisons of subgingival microbial profiles of refractory periodontitis, severe periodontitis, and periodontal health using the human oral microbe identification microarray. J Periodontol 80:1421–1432. doi:10.1902/jop.2009.090185. 19722792 PMC3627366

[11] Nonnenmacher C, Dalpke A, Mutters R, Heeg K. 2004. Quantitative detection of periodontopathogens by real-time PCR. J Microbiol Methods 59:117–125. doi:10.1016/j.mimet.2004.06.006. 15325758

[12] Turng BF, Guthmiller JM, Minah GE, Falkler WA, Jr. 1996. Development and evaluation of a selective and differential medium for the primary isolation of *Peptostreptococcus micros*. Oral Microbiol Immunol 11:356–361. doi:10.1111/j.1399-302x.1996.tb00194.x. 9028263

[13] Rocas IN, Siqueira JF, Jr. 2008. Root canal microbiota of teeth with chronic apical periodontitis. J Clin Microbiol 46:3599–3606. doi:10.1128/JCM.00431-08. 18768651 PMC2576597

[14] Gomes BP, Berber VB, Kokaras AS, Chen T, Paster BJ. 2015. Microbiomes of endodontic-periodontal lesions before and after chemomechanical preparation. J Endod 41:1975–1984. doi:10.1016/j.joen.2015.08.022. 26521147 PMC7061340

[15] Siqueira JF, Jr, Rocas IN. 2013. Microbiology and treatment of acute apical abscesses. Clin Microbiol Rev 26:255–273. doi:10.1128/CMR.00082-12. 23554416 PMC3623375

[16] Neilands J, Davies JR, Bikker FJ, Svensater G. 2019. *Parvimonas micra* stimulates expression of gingipains from *Porphyromonas gingivalis* in multi-species communities. Anaerobe 55:54–60. doi:10.1016/j.anaerobe.2018.10.007. 30359695

[17] van Dalen PJ, van Deutekom-Mulder EC, de Graaff J, van Steenbergen TJ. 1998. Pathogenicity of *Peptostreptococcus micros* morphotypes and *Prevotella* species in pure and mixed culture. J Med Microbiol 47:135–140. doi:10.1099/00222615-47-2-135. 9879956

[18] Cogoni V, Morgan-Smith A, Fenno JC, Jenkinson HF, Dymock D. 2012. *Treponema denticola* chymotrypsin-like proteinase (CTLP) integrates spirochaetes within oral microbial communities. Microbiology (Reading) 158:759–770. doi:10.1099/mic.0.055939-0. 22313692 PMC4851253

[19] Horiuchi A, Kokubu E, Warita T, Ishihara K. 2020. Synergistic biofilm formation by *Parvimonas micra* and *Fusobacterium nucleatum*. Anaerobe 62:102100. doi:10.1016/j.anaerobe.2019.102100. 31521732

[20] Liu K, Hou BX. 2018. The regulation of DLTA gene in bacterial growth and biofilm formation by *Parvimonas micra*. Eur Rev Med Pharmacol Sci 22:4033–4044. doi:10.26355/eurrev_201807_15390. 30024592

[21] Doan N, Contreras A, Flynn J, Morrison J, Slots J. 1999. Proficiencies of three anaerobic culture systems for recovering periodontal pathogenic bacteria. J Clin Microbiol 37:171–174. doi:10.1128/JCM.37.1.171-174.1999. 9854085 PMC84198

[22] Jin DJ, Gross CA. 1988. Mapping and sequencing of mutations in the *Escherichia coli rpoB* gene that lead to rifampicin resistance. J Mol Biol 202:45–58. doi:10.1016/0022-2836(88)90517-7. 3050121

[23] Telenti A, Imboden P, Marchesi F, Lowrie D, Cole S, Colston MJ, Matter L, Schopfer K, Bodmer T. 1993. Detection of rifampicin-resistance mutations in *Mycobacterium tuberculosis*. Lancet 341:647–650. doi:10.1016/0140-6736(93)90417-f. 8095569

[24] Knapp S, Brodal C, Peterson J, Qi F, Kreth J, Merritt J. 2017. Natural competence is common among clinical isolates of *Veillonella parvula* and is useful for genetic manipulation of this key member of the oral microbiome. Front Cell Infect Microbiol 7:139. doi:10.3389/fcimb.2017.00139. 28473967 PMC5397411

[25] Zou Z, Qin H, Brenner AE, Raghavan R, Millar JA, Gu Q, Xie Z, Kreth J, Merritt J. 2018. LytTR regulatory systems: a potential new class of prokaryotic sensory system. PLoS Genet 14:e1007709. doi:10.1371/journal.pgen.1007709. 30296267 PMC6193735

[26] Kung SH, Retchless AC, Kwan JY, Almeida RP. 2013. Effects of DNA size on transformation and recombination efficiencies in *Xylella fastidiosa*. Appl Environ Microbiol 79:1712–1717. doi:10.1128/AEM.03525-12. 23315739 PMC3591940

[27] Shen P, Huang HV. 1986. Homologous recombination in *Escherichia coli*: dependence on substrate length and homology. Genetics 112:441–457. doi:10.1093/genetics/112.3.441. 3007275 PMC1202756

[28] Clark AJ, Margulies AD. 1965. Isolation and characterization of recombination-deficient mutants of *Escherichia Coli* K12. Proc Natl Acad Sci USA 53:451–459. doi:10.1073/pnas.53.2.451. 14294081 PMC219534

[29] Lusetti SL, Wood EA, Fleming CD, Modica MJ, Korth J, Abbott L, Dwyer DW, Roca AI, Inman RB, Cox MM. 2003. C-terminal deletions of the *Escherichia coli* RecA protein. Characterization of in vivo and in vitro effects. J Biol Chem 278:16372–16380. doi:10.1074/jbc.M212917200. 12598539

[30] Merritt J, Senpuku H, Kreth J. 2016. Let there be bioluminescence: development of a biophotonic imaging platform for in situ analyses of oral biofilms in animal models. Environ Microbiol 18:174–190. doi:10.1111/1462-2920.12953. 26119252 PMC5050008

[31] Xie Z, Qi F, Merritt J. 2013. Development of a tunable wide-range gene induction system useful for the study of streptococcal toxin-antitoxin systems. Appl Environ Microbiol 79:6375–6384. doi:10.1128/AEM.02320-13. 23934493 PMC3811191

[32] Topp S, Reynoso CM, Seeliger JC, Goldlust IS, Desai SK, Murat D, Shen A, Puri AW, Komeili A, Bertozzi CR, Scott JR, Gallivan JP. 2010. Synthetic riboswitches that induce gene expression in diverse bacterial species. Appl Environ Microbiol 76:7881–7884. doi:10.1128/AEM.01537-10. 20935124 PMC2988590

[33] Pedrolli D, Langer S, Hobl B, Schwarz J, Hashimoto M, Mack M. 2015. The ribB FMN riboswitch from *Escherichia coli* operates at the transcriptional and translational level and regulates riboflavin biosynthesis. FEBS J 282:3230–3242. doi:10.1111/febs.13226. 25661987

[34] van Opijnen T, Lazinski DW, Camilli A. 2015. Genome-wide fitness and genetic interactions determined by Tn-seq, a high-throughput massively parallel sequencing method for microorganisms. Curr Protoc Microbiol 106:17.16.1–17.16.24. doi:10.1002/0471142727.mb0716s106. PMC4696536 25641100

[35] Ding Q, Tan KS. 2017. Himar1 transposon for efficient random mutagenesis in *Aggregatibacter actinomycetemcomitans*. Front Microbiol 8:1842. doi:10.3389/fmicb.2017.01842. 29018421 PMC5622930

[36] Lampe DJ, Churchill ME, Robertson HM. 1996. A purified mariner transposase is sufficient to mediate transposition in vitro. EMBO J 15:5470–5479. doi:10.1002/j.1460-2075.1996.tb00930.x. 8895590 PMC452289

[37] Brook I. 1988. Recovery of anaerobic bacteria from clinical specimens in 12 years at two military hospitals. J Clin Microbiol 26:1181–1188. doi:10.1128/jcm.26.6.1181-1188.1988. 3384929 PMC266558

[38] Murdoch DA, Mitchelmore IJ, Tabaqchali S. 1988. *Peptostreptococcus micros* in polymicrobial abscesses. Lancet 1:594. doi:10.1016/S0140-6736(88)91393-1. 2894531

[39] Kim EY, Baek YH, Jung DS, Woo KS. 2019. Concomitant liver and brain abscesses caused by *Parvimonas micra*. Korean J Gastroenterol 73:230–234. doi:10.4166/kjg.2019.73.4.230. 31030461 PMC12285808

[40] Yoo LJH, Zulkifli MD, O'Connor M, Waldron R. 2019. *Parvimonas micra* spondylodiscitis with psoas abscess. BMJ Case Rep 12:e232040. doi:10.1136/bcr-2019-232040. PMC6887430 31748364

[41] Drewes JL, White JR, Dejea CM, Fathi P, Iyadorai T, Vadivelu J, Roslani AC, Wick EC, Mongodin EF, Loke MF, Thulasi K, Gan HM, Goh KL, Chong HY, Kumar S, Wanyiri JW, Sears CL. 2017. High-resolution bacterial 16S rRNA gene profile meta-analysis and biofilm status reveal common colorectal cancer consortia. NPJ Biofilms Microbiomes 3:34. doi:10.1038/s41522-017-0040-3. 29214046 PMC5707393

[42] Purcell RV, Visnovska M, Biggs PJ, Schmeier S, Frizelle FA. 2017. Distinct gut microbiome patterns associate with consensus molecular subtypes of colorectal cancer. Sci Rep 7:11590. doi:10.1038/s41598-017-11237-6. 28912574 PMC5599497

[43] Zhang M, Lv Y, Hou S, Liu Y, Wang Y, Wan X. 2021. Differential mucosal microbiome profiles across stages of human colorectal cancer. Life (Basel) 11:831. doi:10.3390/life11080831. 34440574 PMC8401903

[44] Sun J, Tang Q, Yu S, Xie M, Xie Y, Chen G, Chen L. 2020. Role of the oral microbiota in cancer evolution and progression. Cancer Med 9:6306–6321. doi:10.1002/cam4.3206. 32638533 PMC7476822

[45] Dubnau D, Blokesch M. 2019. Mechanisms of DNA uptake by naturally competent bacteria. Annu Rev Genet 53:217–237. doi:10.1146/annurev-genet-112618-043641. 31433955

[46] Chen I, Dubnau D. 2004. DNA uptake during bacterial transformation. Nat Rev Microbiol 2:241–249. doi:10.1038/nrmicro844. 15083159

[47] Blakesley RW, Dodgson JB, Nes IF, Wells RD. 1977. Duplex regions in “single-stranded” phiX174 DNA are cleaved by a restriction endonuclease from *Haemophilus aegyptius*. J Biol Chem 252:7300–7306. doi:10.1016/S0021-9258(19)66969-3. 71298

[48] Boyer HW. 1974. Restriction and modification of DNA: enzymes and substrates. Introductory remarks. Fed Proc 33:1125–1127. 4599005

[49] Renkawitz J, Lademann CA, Jentsch S. 2014. Mechanisms and principles of homology search during recombination. Nat Rev Mol Cell Biol 15:369–383. doi:10.1038/nrm3805. 24824069

[50] Huang M, Liu M, Huang L, Wang M, Jia R, Zhu D, Chen S, Zhao X, Zhang S, Gao Q, Zhang L, Cheng A. 2021. The activation and limitation of the bacterial natural transformation system: the function in genome evolution and stability. Microbiol Res 252:126856. doi:10.1016/j.micres.2021.126856. 34454311

[51] Dutra BE, Sutera VA, Jr, Lovett ST. 2007. RecA-independent recombination is efficient but limited by exonucleases. Proc Natl Acad Sci USA 104:216–221. doi:10.1073/pnas.0608293104. 17182742 PMC1765439

[52] Escapa IF, Chen T, Huang Y, Gajare P, Dewhirst FE, Lemon KP. 2018. New insights into human nostril microbiome from the Expanded Human Oral Microbiome Database (eHOMD): a resource for the microbiome of the human aerodigestive tract. mSystems 3. doi:10.1128/mSystems.00187-18. PMC6280432 30534599

[53] Cho E, Park SN, Lim YK, Shin Y, Paek J, Hwang CH, Chang YH, Kook JK. 2015. *Fusobacterium hwasookii* sp. nov., isolated from a human periodontitis lesion. Curr Microbiol 70:169–175. doi:10.1007/s00284-014-0692-7. 25257648

[54] Thakker-Varia S, Ranzini AC, Dubin DT. 1985. Ribosomal RNA methylation in Staphylococcus aureus and *Escherichia coli*: effect of the “MLS” (erythromycin resistance) methylase. Plasmid 14:152–161. doi:10.1016/0147-619x(85)90075-7. 3906713

[55] Trieu-Cuot P, Courvalin P. 1983. Nucleotide sequence of the *Streptococcus faecalis* plasmid gene encoding the 3'5"-aminoglycoside phosphotransferase type III. Gene 23:331–341. doi:10.1016/0378-1119(83)90022-7. 6313476

